# Altered Brain Cholinergic and Synaptic Markers in Obese Zucker Rats

**DOI:** 10.3390/cells10102528

**Published:** 2021-09-24

**Authors:** Ilenia Martinelli, Daniele Tomassoni, Proshanta Roy, Francesco Amenta, Seyed Khosrow Tayebati

**Affiliations:** 1 School of Pharmacy, University of Camerino, 62032 Camerino, Italy; ilenia.martinelli@unicam.it (I.M.); francesco.amenta@unicam.it (F.A.); 2 School of Biosciences and Veterinary Medicine, University of Camerino, 62032 Camerino, Italy; daniele.tomassoni@unicam.it (D.T.); proshanta.roy@unicam.it (P.R.)

**Keywords:** brain, cholinergic system, synaptic transmission, metabolic syndrome, obesity

## Abstract

The association between obesity and loss of cognitive performance has been recognized. Although there are data regarding the metabolic alterations in obese conditions and the development of neuroinflammation, no clear evidence concerning obesity-related cholinergic and synaptic impairments in the frontal cortex and hippocampus has been reported yet. Here, we investigate different cholinergic and synaptic markers in 12-, 16-, and 20-week-old obese Zucker rats (OZRs) compared with lean littermate rats (LZRs), using immunochemical and immunohistochemical analysis. Consequently, OZRs showed body weight gain, hypertension, and dysmetabolism. In 20-week-old OZRs, the reduction of vesicular acetylcholine transporter (VAChT) and alpha7 nicotinic acetylcholine receptors (α7nAChR) occurred both in the frontal cortex and in the hippocampus, suggesting a cognitive dysfunction due to obesity and aging. Among the muscarinic receptors analyzed, the level of expression of type 1 (mAChR1) was lower in the hippocampus of the older OZRs. Finally, we showed synaptic dysfunctions in OZRs, with a reduction of synaptophysin (SYP) and synaptic vesicle glycoprotein 2B (SV2B) in 20-week-old OZRs, both in the frontal cortex and in the hippocampus. Taken together, our data suggest specific alterations of cholinergic and synaptic markers that can be targeted to prevent cognitive deficits related to obesity and aging.

## 1. Introduction

Nowadays, obesity and obesity-related disorders have become widespread conditions. Obesity and the closely related metabolic syndrome (MetS) cause a considerable risk of developing type 2 diabetes mellitus (T2DM), cardiovascular disease (CVD), and other complications [1,2,3]. The major driver of the increasing obesity and diabetes epidemic is the current obesogenic environment, proffering high-calorie foods and physical inactivity. However, not everyone exposed to this environment gains weight or develops T2DM. Indeed, a genetic predisposition or heritability is reported to contribute to obesity and T2DM [4].

Studies have explored biological mechanisms to explain the negative effects of a high-fat diet (HFD) on cognitive performance. Among them, insulin resistance, inflammation, oxidative stress, altered membrane functioning, and vascularization represent the most documented [5,6,7]. An HFD is commonly used to study obesity in mice, and neural inflammation can be assessed even before substantial weight gain [8,9]. In these murine models of diet-induced obesity (DIO), the increased fatty acid (FA) intake increases the activation of immune cells and the inflammatory response in different organs [10]. Briefly, the binding of FAs to Toll-like receptor 4 (TLR4) activates two different transcription factors, nuclear factor κB, (NF-κB) and activator protein 1 (AP-1), that in turn upregulate the expression of proinflammatory mediators, such as cytokines and chemokines [11]. In DIO animals, we have confirmed the presence of hyperglycemia, insulin resistance, and hypertension, accompanied by astrogliosis, microglial activation, and endothelial inflammation in the frontal cortex and in the hippocampus [12]. Among various animal models that have been developed and routinely used to study the pathogenesis and mechanisms of obesity/T2DM, the non-leptin-deficient DIO mice or rats and genetically obese mice or rats, i.e., leptin-receptor-deficient obese (*fa*/*fa)* Zucker rats (OZRs), remain the most widely used experimental models.

Preclinical genetic models such as *ob*/*ob* and *db*/*db* mice or the OZRs were pivotal in unraveling many signaling pathways involved in obesity. As well as in the DIO model, we have previously reported in OZRs blood–brain barrier (BBB) alterations, neuronal loss, and gliosis both in the frontal cortex and in the hippocampus [13]. The behavioral tests revealed cognitive alterations in older OZRs as well as in DIO rats [12,14].

The cholinergic system has been revealed to be involved in the regulation of food intake and energy expenditure. Moreover, physical exercise promotes a reduction of fat pads and body mass by increasing energy expenditure but also influences the cholinergic system and synaptic markers [15,16]. Indeed, the beneficial effects of physical exercise on cognitive functions have been well documented in the studies of both rodents and humans [17]. The brain’s cholinergic signaling and the *vagus* nerve have a crucial role in the regulation of metabolic homeostasis and the immune function. Studies supported the therapeutic efficacy of cholinergic stimulation in alleviating obesity-associated metabolic derangements and neuroinflammation [18,19,20,21,22]. The mechanisms of the inflammatory reflex include alpha7 nicotinic acetylcholine receptor (α7nAChR)-mediated signaling in its efferent arm. Cholinergic drugs, including α7nAChR agonists and acetylcholinesterase (AChE) inhibitors, have also been shown to be cognitive enhancers and to reduce inflammation and metabolic imbalances in obesity and in MetS [22,23]. For instance, donepezil reversed obesity-related central inflammation and oxidative damage and improved memory impairments in HFD-fed mice [24]. In addition, galantamine showed anti-inflammatory and beneficial metabolic effects in patients with MetS [25]. The muscarinic acetylcholine receptors (mAChRs) were also found to be strongly influenced by obesity in DIO rats [26].

Not only the cholinergic parameters seem to be altered because of the obese condition but synaptic marker expression in prefrontal and perirhinal cortex also decreased in DIO rats, accompanied by decreased dendritic spine density and finally cognitive deficits [27]. Microglial morphology was also changed in the prefrontal cortex. Synaptic proteins, including vesicle-associated with the pre- and postsynaptic membrane proteins, are closely related to cognitive function. Previous studies have shown that the loss of synapses in the brain tissues of patients with Alzheimer’s disease (AD) was associated with cognitive impairment [28,29]. Synaptophysin (SYP), a specific presynaptic marker of vesicles that reflects the density and distribution of synapses, serves a crucial role in neural plasticity, influencing the synaptic structure and mediating neurotransmitter release via phosphorylation [30].

Although there are numerous data regarding the metabolic alterations in obese conditions and the development of neuroinflammation [8,9,12,14,31], no mechanism has been presented concerning obesity-related cholinergic and synaptic impairments in the brain. Therefore, this study was designed to investigate whether the memory and learning impairments in older OZRs [14] were also related to cerebral cholinergic and synaptic alterations, specifically identifying the markers that were implicated. The investigation was carried out in brain areas, in which cholinergic neurotransmission is widely represented: the frontal cortex, especially the motor region, and the hippocampus, which is involved in learning and memory tasks [14]. The OZR is a model of MetS for the concomitant manifestation of obesity, hyperglycemia, hyperinsulinemia, hyperlipidemia, and moderate hypertension [13,14,32], compared to littermate lean Zucker rats (LZRs).

## 2. Materials and Methods

### 2.1. Ethical Animal Handling

Experimental procedures were carried out according to the Institutional Guidelines and complied with the Italian Ministry of Health (D. Lgs. 116/92–Art. 7) (Prot. N. 6198/2011) and associated guidelines from European Communities Council Directive (n. 86/609/CEE) governing animal welfare and protection.

### 2.2. Animals

Male OZRs (*n* = 18) and their littermate lean Zucker rats (LZRs) (*n* = 18) were purchased from Harlan (Italy). They were grouped into six animals for each strain based on the age at sacrifice, performed at 12, 16, and 20 weeks of age, as previously described [14,32]. Based on previous studies [13,33,34,35], the age at sacrifice and the number of animals for each experimental group was identified. Starting from the 10th week of age, the rats were housed in one for cage and maintained on a 12 h light/dark cycle (lights on at 7 a.m.). They were fed with standard diet (Mucedola 4RF18 mice and rats long-term maintenance, containing 16% protein, 2.5% fat, and 7.5% max fiber and other nutritional additives) with *ad libitum* access to food and water. Body weights were measured daily. Values of systolic blood pressure were recorded once a week by tail-cuff methods using an electronic sphygmomanometer (B3Plus, GIMA, Italy) on conscious rats [14,32].

### 2.3. Biochemical Analysis

Before sacrifice, blood withdrawals were performed from the tail vein in fasted rats. Blood samples were collected into tubes with L-heparin. Serum was separated by centrifugation of samples at 3000 rpm for 10 min to measure the blood glucose, insulin, triglycerides, and total cholesterol, as previously described [14,32].

### 2.4. Tissue Handling

The brains were carefully removed and divided into two hemispheres. In the right hemisphere, the frontal cortex and hippocampus were collected and frozen at −80 °C for Western blot analysis, while the left one was fixed in 4% paraformaldehyde in 0.1 M pH 7.4 phosphate-buffered saline (PBS) and embedded in paraffin wax for immunohistochemical analysis [14].

### 2.5. Western Blot (WB) and Quantification

Protein lysate was obtained by homogenizing brain areas (100 ± 2 mg) in a Mixer Mill MM300 (Qiagen, Hilden, Germany) for 10 min, using lysis buffer. Next, 40 µg of proteins were separated on SDS polyacrylamide gels, transferred onto nitrocellulose membranes, and blotted with the specific antibodies as previously described [14,32]. After incubation with blocking solution (5% BSA in PBS 0.1% Tween-20), membranes were incubated at 4 °C overnight with the primary antibodies as detailed in Table 1. The specificity of immune reaction was assessed using antibodies pre-adsorbed with peptides employed for generating them [36,37]. The blots were then incubated for 1 h at room temperature with the corresponding horseradish peroxidase (HRP)-conjugated secondary antibodies (BETHYL Laboratories, Inc., Montgomery, TX, USA, dilution 1:5000). LiteAblot PLUS or Turbo kits (EuroClone, Milan, Italy) were used as the detection system followed by densitometric analysis carried out by Quantity One software of the ChemiDoc apparatus (Bio-Rad, Hercules, CA, USA), using GAPDH as the loading control. Blots are representative of one of three experimental sessions.

### 2.6. Immunohistochemistry (IHC) and Image Analysis 

The paraffin-embedded tissue from each rat was sectioned at 10 μm with a microtome. Five groups of ten consecutive sagittal sections were attached to poly-l-lysine-coated slides. As previously described [14] the first of each group of ten consecutive sections was stained with a 0.5% cresyl violet to highlight the possible morphological alterations. The others were processed independently for immunohistochemistry using different antibodies at various dilutions in PBS + TritonX-100 0.3% (PBS-T), as detailed in Table 1. Optimal antibody concentration and specificity of the antibodies were established in a series of preliminary experiments in which parallel control slides were exposed to the same antibody absorbed with the blocking peptide for 3 h at 4 °C [36,37]. The immune reaction was revealed by exposing slides for 30 min at 25 °C (dilution 1:200 in PBS-T) to the specific biotinylated secondary antibodies (BETHYL Laboratories, Inc., Montgomery, TX, USA). The sections were incubated with an avidin–biotin kit (Vector Laboratories, Inc., Burlingame, CA, USA, dilution 1:100) using as substrate a 3,3′-diamonobenzidine tetrahydrochloride (DAB) solution (Vector Laboratories, Inc., Burlingame, CA, USA). The sections were observed with a microscope Leica DMR connected by a DS-Ri2 NIKON camera to NIS Elements Nikon image analyzer software (Nikon, Florence, Italy) to record the mean intensities of immune reaction as previously described [14].

### 2.7. Immunofluorescence

For confocal laser microscopy, slides were incubated with vesicular acetylcholine transporter (VAChT) primary antibody (Table 1), followed by incubation with donkey anti-goat Alexa Fluor 488 secondary antibody (1 h at 37 °C) and then counterstained with DAPI (1:100 in PBS-T). Sections were viewed using a Nikon mod. C2 plus Confocal Laser Microscope (Nikon, Corporation, Japan). Representative pictures were captured at 60× magnification, zoom 2×.

### 2.8. Statistical Analysis 

Means of different parameters investigated were calculated from single-animal data, and expressed as the means ± S.E.M. The significance of differences between means was estimated by analysis of variance (ANOVA) followed by the Bonferroni multiple range tests, setting *p* < 0.05 value as a significant difference.

## 3. Results

### 3.1. General and Blood Analysis

The value of body weight, as well as the food intake, were significantly higher in OZRs than in LZRs, starting from 10 weeks of age until 20 weeks of age. Serum analyses showed that glucose and insulin were higher in OZRs than in LZRs in all weeks. Furthermore, triglycerides levels were higher, and total, LDL, and HDL cholesterol increased proportionally to age in the obese animals, indicating a condition of dysmetabolism [14,32]. Moreover, the values of systolic blood pressure were significantly higher starting from 16 weeks of age in OZRs (140.8 ± 5.6 mmHg at 16 weeks, *p* = 0.002 and 137.3 ± 4.2 mmHg at 20 weeks of age, *p* = 0.007 vs. age-matched LZRs).

### 3.2. Cholinergic Marker: Vesicular Acetylcholine Transporter

**Figure 1:**
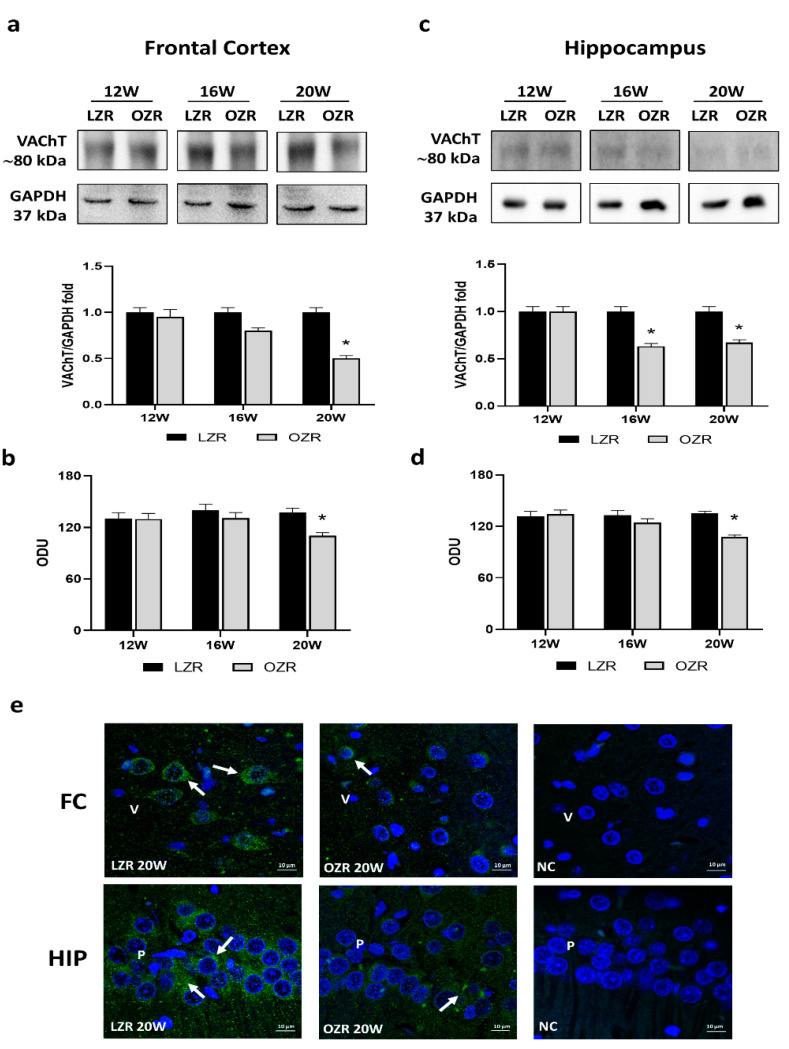
Immunoblotting and immunofluorescence of vesicular acetylcholine transporter (VAChT). Lysates of the frontal cortex (**a**) and hippocampus (**c**) from lean Zucker rats (LZRs) and obese Zucker rats (OZRs) at the age of 12, 16, and 20 weeks were immunoblotted using specific anti-VAChT. Bar graphs indicate the densitometric analysis using LZRs as control, and GAPDH levels were used as a loading control. Blots are representative of one of three separate experiments; intensity values of VAChT immunostaining in the frontal cortex (**b**) and hippocampus (**d**) from LZRs and OZRs at the age of 12, 16, and 20 weeks measured in optical density units (ODUs). Data are mean ± S.E.M. * *p* < 0.05 vs. age-matched LZRs. (**e**) Representative immunofluorescence pictures of 20-week-old LZRs and OZRs in the frontal cortex (FC) and hippocampus (HIP). Arrows indicate VAChT, labeling. V: the fifth layer of the frontal cortex. NC: negative control. P: pyramidal neurons of the hippocampus. 60× magnification zoom 2×. Calibration bar: 10 µm.

Western blot analyses, performed in the frontal cortex (Figure 1a) and the hippocampus (Figure 1c), showed a decrease in the expression of VAChT with a band around 80 kDa, corresponding to its mature glycosylated form [36], in OZRs compared to control LZRs at 20 weeks of age. In the 16-week-old obese phenotype, the expression of VAChT was significantly reduced in the hippocampus (Figure 1c) but not in the frontal cortex (Figure 1a). In line with the Western blot was the immunohistochemistry analysis in which the average intensity values of VAChT were remarkably reduced in 20-week-old OZRs, both in the frontal cortex (Figure 1b) and in the hippocampus (Figure 1d). Immunofluorescent procedures revealed that VAChT labeled the neuronal soma of the pyramidal neurons and along the cholinergic fibers in the frontal cortex and in the CA1 subfield of the hippocampus, as showed by representative pictures (Figure 1e). 

### 3.3. Synaptic Markers

#### 3.3.1. Cholinergic Receptors

**Figure 2:**
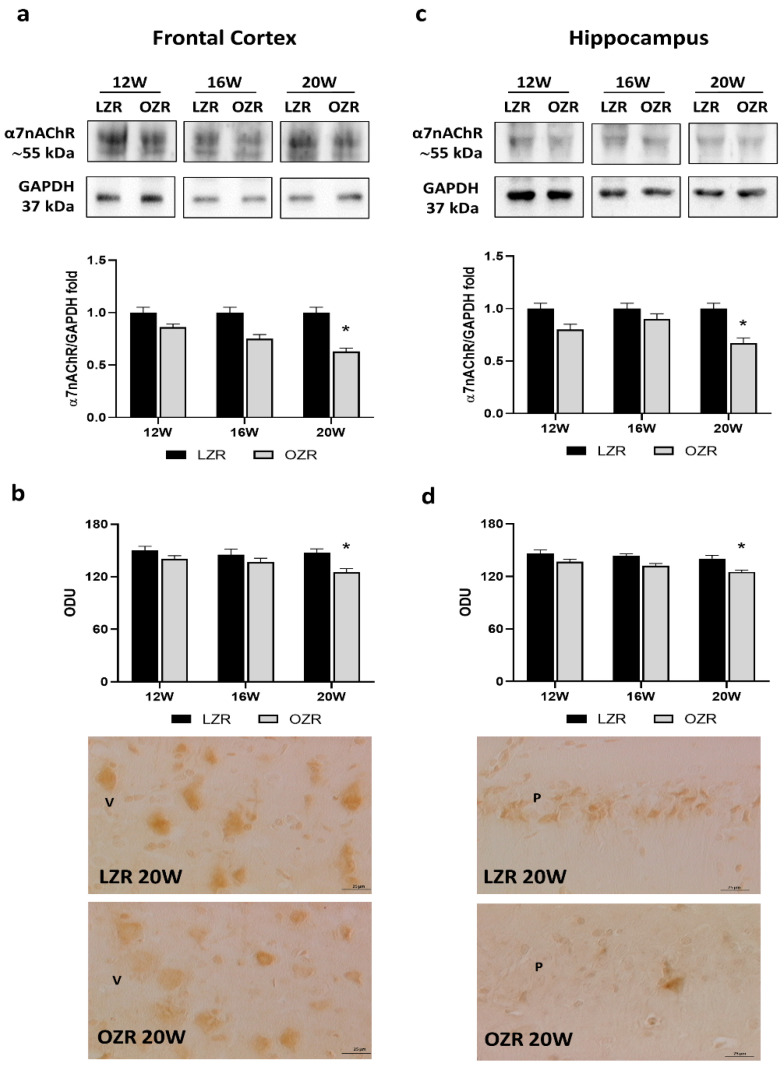
Immunoblotting and immunoreaction of alpha7 nicotinic acetylcholine receptor (α7nAChR). Lysates of frontal cortex (**a**) and hippocampus (**c**) from lean Zucker rats (LZRs) and obese Zucker rats (OZRs) at the age of 12, 16, and 20 weeks were immunoblotted using specific anti-α7nAChR. Bar graphs indicate the densitometric analysis using LZRs as control, and GAPDH levels were used as loading control. Blots are representative of one of three separate experiments; intensity values of α7nAChR immunostaining in the frontal cortex (**b**) and the hippocampus (**d**) from LZRs and OZRs at the age of 12, 16, and 20 weeks measured in optical density units (ODUs). Data are mean ± S.E.M. * *p* < 0.05 vs. age-matched LZR. Representative pictures of 20 weeks old LZR and OZR in frontal cortex (**b**) and hippocampus (**d**). V: the fifth layer of the frontal cortex. P: pyramidal neurons of the hippocampus. 40× magnification. Calibration bar: 25 µm.

Among the nicotinic receptors, the alpha7 subunit (α7nAChR) constitutes one of the predominant nAChR subtypes in the mammalian brain [38] and is widely expressed pre- and postsynaptically also in the hippocampus [39]. Immunochemical analyses for the α7nAChR showed a band around 55 kDa both in the frontal cortex (Figure 2a) and in the hippocampus (Figure 2c). In both these areas, protein quantification demonstrated a reduction of α7nAChR expression in OZRs, in particular at 20 weeks, compared with age-matched lean rats (Figure 2a,c). Immunoreactivity for the nicotinic receptor α7nAChR was localized in the pyramidal neurons in the fifth (V) layer of the frontal cortex (Figure 2b). At the level of the hippocampus, pyramidal neurons were reactive both in the CA1 subfield (Figure 2d) and in subfield CA3. The immunoreaction of α7nAChR was reported to be significantly reduced in older obese rats, both in the frontal cortex (Figure 2b) and in the hippocampus (Figure 2d).

**Figure 3:**
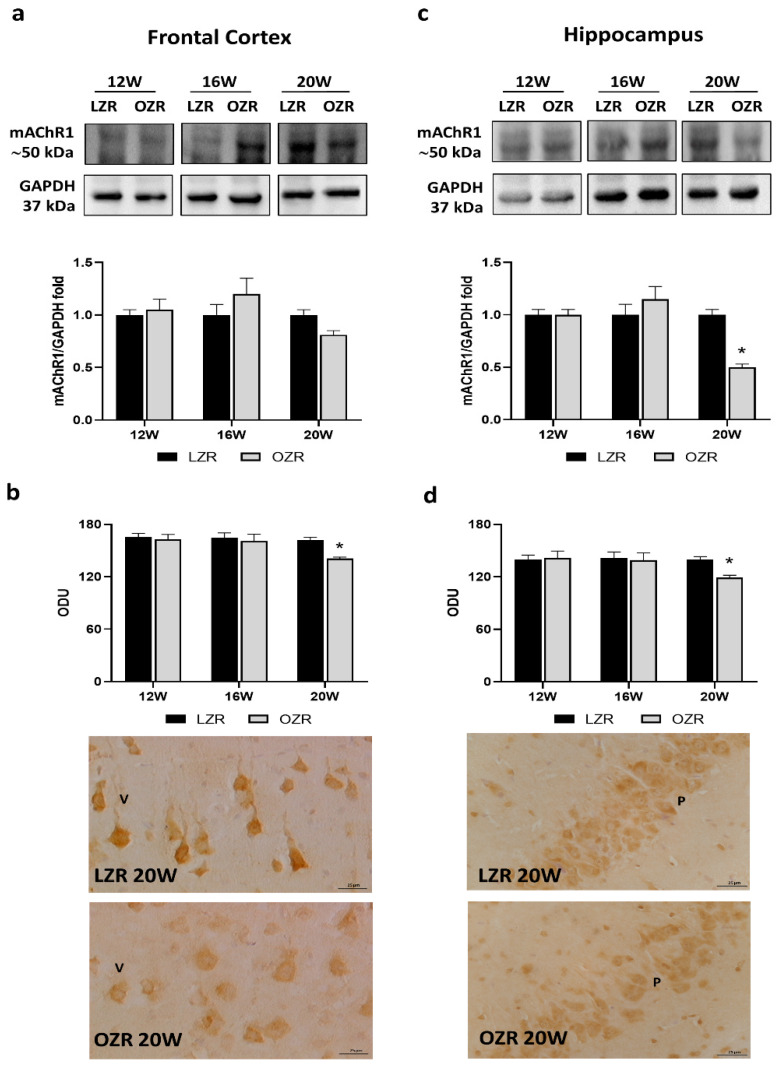
Immunoblotting and immunoreaction of muscarinic acetylcholine receptor subtype 1 (mAChR1). Lysates of the frontal cortex (**a**) and hippocampus (**c**) from lean Zucker rats (LZRs) and obese Zucker rats (OZRs) at the age of 12, 16, and 20 weeks were immunoblotted using specific anti-mAChR1. Bar graphs indicate the densitometric analysis using LZRs as control, and GAPDH levels were used as a loading control. Blots are representative of one of three separate experiments; intensity values of mAChR1 immunostaining in the frontal cortex (**b**) and hippocampus (**d**) from LZRs and OZRs at the age of 12, 16, and 20 weeks measured in optical density units (ODUs). Data are mean ± S.E.M. * *p* < 0.05 vs. age-matched LZR. Representative pictures of 20-week-old LZRs and OZRs in the frontal cortex (**b**) and hippocampus (**d**). V: the fifth layer of the frontal cortex. P: pyramidal neurons of the hippocampus. 40× magnification. Calibration bar: 25 µm.

**Figure 4:**
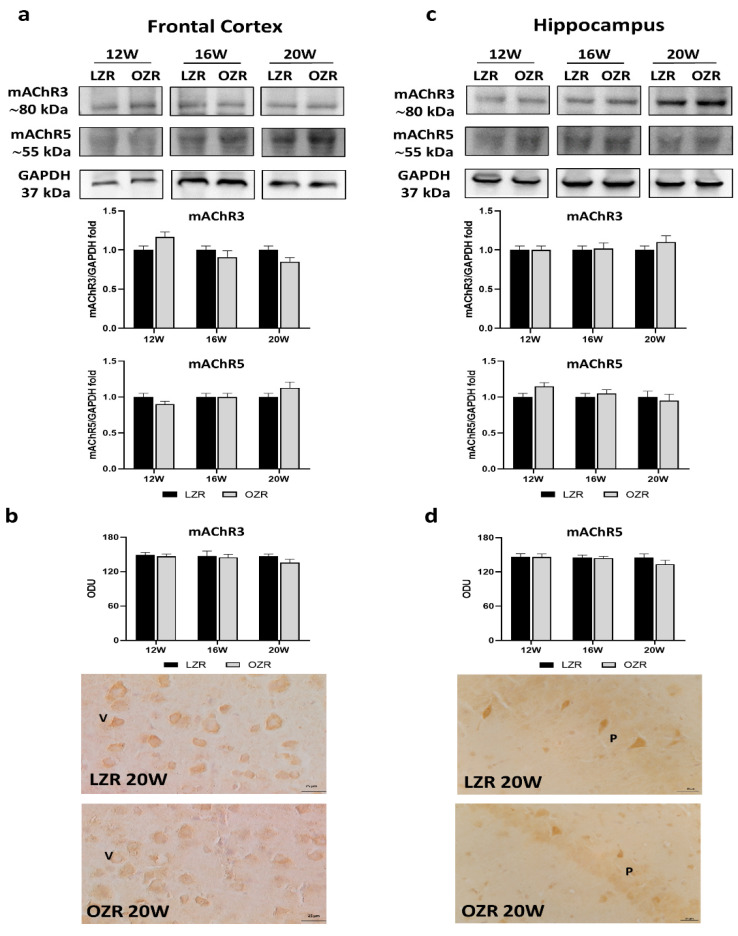
Immunoblotting and immunoreaction of muscarinic acetylcholine receptor subtype 3 (mAChR3) and muscarinic acetylcholine receptor subtype 5 (mAChR5). Lysates of the frontal cortex (**a**) and hippocampus (**c**) from lean Zucker rats (LZRs) and obese Zucker rats (OZRs) at the age of 12, 16, and 20 weeks were immunoblotted using specific anti-mAChR3 and anti-mAChR5. Bar graphs indicate the densitometric analysis using LZRs as control, and GAPDH levels were used as a loading control. Blots are representative of one of three separate experiments; intensity values of mAChR3 immunostaining in the frontal cortex (**b**) and mAChR5 in the hippocampus (**d**) from LZRs and OZRs at the age of 12, 16, and 20 weeks measured in optical density units (ODUs). Data are mean ± S.E.M. Representative pictures of 20-week-old LZRs and OZRs in frontal cortex for mAChR3 (**b**) and hippocampus for mAChR5 (**d**). V: the fifth layer of the frontal cortex. P: pyramidal neurons of the hippocampus. 40× magnification. Calibration bar: 25 µm.

We decided to explore the expressions of the following muscarinic receptors: mAChR1, mAChR3, and mAChR5, based on the most abundant localization both in the cerebral cortex and hippocampus [40,41], and the fact that all they are expressed predominantly postsynaptically, G_q_ coupled, and stimulated by the phospholipase C (PLC) and inositol trisphosphate (IP3) signal transduction pathways to increase cytosolic calcium levels [42]. The results showed that, among the muscarinic receptors analyzed (Figure 3 and Figure 4), only mAChR1 was reduced in obese conditions (Figure 3a–d).

Western blot results for the mAChR1 showed a band around 50 kDa in the frontal cortex (Figure 3a) and the hippocampus (Figure 3c). In 20-week-old OZRs, the expression of mAChR1 was significantly reduced compared to that in age-matched LZRs in the hippocampus (Figure 3c) but not in the frontal cortex (Figure 3a). In addition, the immunohistochemistry analysis confirmed a lower mAChR1 immunoreaction at 20 weeks of age in obese conditions compare to that in control lean rats in both the areas investigated (Figure 3b,d).

mAChR3 and mAChR5 receptors were expressed at around 80 and 55 kDa, respectively (Figure 4a,c). Neither the levels of mAChR3 and mAChR5 (Figure 4a,c) nor their immunoreaction (Figure 4b,d) were significantly different among the animals. Indeed, similar values were reported between the age-matched opposite groups (Figure 4a–d). Both these receptor subtypes were present in the fifth layer of the frontal cortex, and in the CA1 and CA2 subfields of the hippocampus.

#### 3.3.2. Synaptic Vesicle Glycoproteins

**Figure 5:**
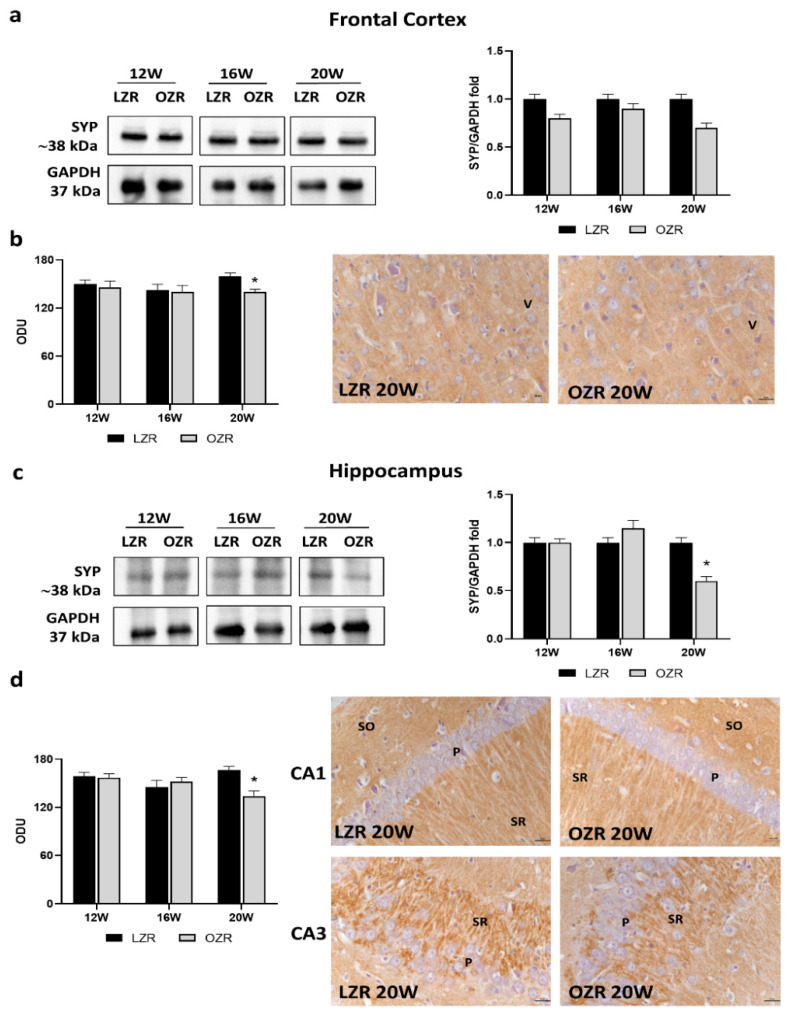
Immunoblotting and immunoreaction of synaptophysin (SYP). Lysates of the frontal cortex (**a**) and hippocampus (**c**) from lean Zucker rats (LZRs) and obese Zucker rats (OZRs) at the age of 12, 16, and 20 weeks were immunoblotted using specific anti-SYP. Bar graphs indicate the densitometric analysis using LZRs as control, and GAPDH levels were used as the loading control. Blots are representative of one of three separate experiments; intensity values of SYP immunostaining in the frontal cortex (**b**) and hippocampus (**d**) from LZRs and OZRs at the age of 12, 16, and 20 weeks measured in optical density units (ODUs). Data are mean ± S.E.M. * *p* < 0.05 vs. age-matched LZRs. Representative pictures of 20-week-old LZRs and OZRs in frontal cortex (**b**) and hippocampus (**d**). V: the fifth layer of the frontal cortex. P: pyramidal neurons in the CA1 and CA3 subfields of the hippocampus. SO: *stratum oriens*. SR: *stratum radiatum*. 40× magnification. Calibration bar: 25 µm.

As an abundant synaptic marker, SYP was explored (Figure 5). The Western blot results did not show statistical differences in SYP levels in the obese condition compared to the lean one in the frontal cortex (Figure 5a). Instead, the quantification of SYP immunoreaction was significantly reduced only in 20-week-old OZRs in comparison with the age-matched controls as demonstrated by representative pictures (Figure 5b). Moreover, in the hippocampus, the SYP expression (Figure 5c), as well as the immunoreaction localized in CA1 and CA3 (Figure 5d), was remarkably reduced in the older OZRs compared to that in controls.

**Figure 6:**
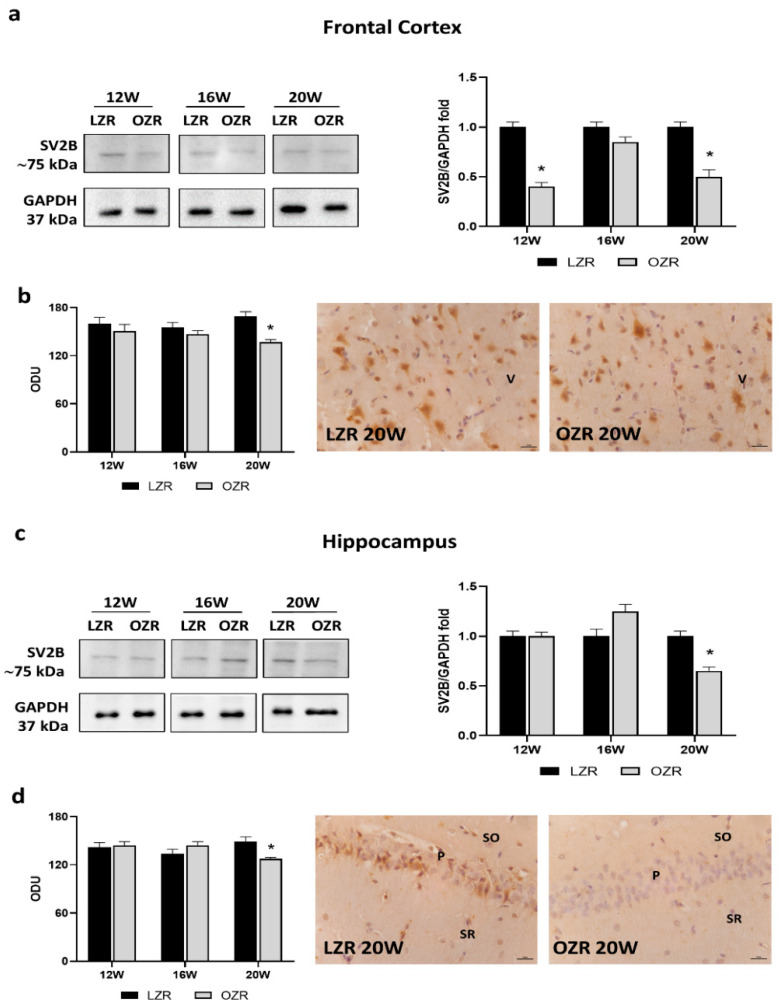
Immunoblotting and immunoreaction of synaptic vesicle glycoprotein 2B (SV2B). Lysates of the frontal cortex (**a**) and hippocampus (**c**) from lean Zucker rats (LZRs) and obese Zucker rats (OZRs) at the age of 12, 16, and 20 weeks were immunoblotted using specific anti-SV2B. Bar graphs indicate the densitometric analysis using LZRs as control, and GAPDH levels were used as the loading control. Blots are representative of one of three separate experiments; intensity values of SV2B immunostaining in the frontal cortex (**b**) and hippocampus (**d**) from LZRs and OZRs at the age of 12, 16, and 20 weeks measured in optical density units (ODUs). Data are mean ± S.E.M. * *p* < 0.05 vs. age-matched LZRs. Representative pictures of 20-week-old LZRs and OZRs in the frontal cortex (**b**) and hippocampus (**d**). V: the fifth layer of the frontal cortex. P: pyramidal neurons in the CA1 subfield of the hippocampus. SO: *stratum oriens*. SR: *stratum radiatum*. 40× magnification. Calibration bar: 25 µm.

Among the presynaptic vesicle proteins analyzed, i.e., synaptic vesicle glycoproteins 2A and 2C (SVA and SVC, respectively) (Figure S1), only synaptic vesicle glycoprotein 2B (SV2B) showed alterations related to obesity and age (Figure 6). Indeed, results from Western blot and immunohistochemistry showed significantly reduced SV2B levels in the frontal cortex in 20-week-old OZRs compared to that in the lean ones (Figure 6a,b). Moreover, in the hippocampus, the SV2B expression (Figure 6c), as well as the immunoreaction localized in CA1 (Figure 6d), was remarkably reduced in the older OZRs compared to that in controls.

## 4. Discussion

Obesity is a complex disorder connected with several physiological abnormalities that arise from excessive fat tissue accumulation [43]. Different studies showed that obesity related to an HFD impaired learning and memory in rodents, suggesting a strong correlation between obesity and cognitive dysfunction [12,27,44,45,46,47,48,49,50].

Several neurotransmitters including acetylcholine (ACh) have been implicated in the regulation of food intake and obesity [51,52]. In the brain region of Zucker fatty rats analyzed by [53], ACh content showed a lower level than that of the lean rats. In these animals, the activities of AChE were found to be significantly lower than those in the lean rats in all the brain areas, except in the striatum and medulla oblongata, where it was significantly reduced [53]. On the contrary, significantly higher AChE activity was seen in the cerebral cortex, cerebellum, midbrain, thalamus, and hypothalamus of 14-week-old OZRs than in their lean littermates [54]. Meanwhile, choline acetyltransferase (ChAT) activity was lower in the cerebellum, pons, and cerebral cortex, while a significant increase in ChAT activity was found in the thalamus and hypothalamus [54]. Thus, the diencephalon of the OZRs showed a significant increase in both ChAT and AChE activities, which may reflect an increase in the ACh turnover rate. It was postulated that the increase in the turnover rate of ACh was probably a cause of obesity rather than a consequence of obesity [54]. From these controversial and complex data, it was concluded that obesity could be associated with changes in the enzymes activities of the brain cholinergic system also depending on the brain regions [54]. To date, no data have been published yet regarding cholinergic transporter and receptors and synaptic markers in OZRs. The availability of genetically obese rats with known changes in the brain neurochemistry provided an excellent model to study obesity and cholinergic as well as synaptic function in the frontal cortex and hippocampus.

The obese rats (*fa*/*fa*) present dysfunctions in the CNS [14,55]. The current study shows a reduction of VAChT and α7nAChR expressions both in the frontal cortex and in the hippocampus of 20-week-old OZRs. Indeed, VAChT and α7nAChR are considered pro-cognitive elements, directly involved in learning and memory, as well as in the pathology of neurodegenerative and cerebrovascular diseases [56,57,58,59]. This may justify previous behavioral tests that revealed, in 20-week-old OZRs, anxiety-like behavior compared to age-matched LZRs. In addition, the reduced retention latency time in the emotional learning task also confirmed cognitive impairment in OZRs [14].

Nowadays, it is well recognized that nAChRs are expressed not only on neurons but also in microglia [60] and astrocytes [61]. Moreover, their responses are often mediated specifically by α7nAChRs. Among the responses, its regulation of the cholinergic anti-inflammatory pathway [62,63] is gaining great attention. Studies have revealed that the activation of α7nAChR in astrocytes and microglia can induce anti-inflammatory effects through the downregulation of pro-inflammatory cytokine production [59,64,65]. Thus, we can speculate that the astrogliosis, reactive microglia, and vascular inflammation, characterized by the increase of intercellular adhesion molecule-1 (ICAM-1) and vascular cell adhesion molecule-1 (VCAM-1) expressions in the brain of older OZRs [14,66], may be related to the low expression of α7nAChR. Among the mechanisms, neuroinflammation with the increase of cytokines and changes in membrane fluidity and, above all, the disruption of the BBB is the most accredited [44,46]. Local and systemic inflammation, induced by obesity or T2DM, has been linked to central disorders, such as depression, and neurodegenerative diseases such as AD, because of BBB breakdown, decreased removal of waste, and increased infiltration of immune cells [8,9,21,31]. This, in turn, leads to cognitive impairment and disruption of neuronal and glial cells, triggering hormonal dysfunction and amplified immune sensitivity, depending on the affected brain areas (hippocampus, cortex, brainstem, or amygdala) [8,9,67].

Moreover, a high-calorie diet could be involved in the alterations of the cholinergic system with the modulation of mAChRs [26]. Following the results carried out in DIO rats [26], we found that the mAChR1, but not mAChR5, was significantly reduced in the hippocampus of 20-week-old OZRs. Even though downregulation of mAChR3 in the hippocampus of DIO animals has been reported [26], here we did not find any differences either in the hippocampus or in the frontal cortex. Taken together, these results indicate a differential modulation of mAChR1, mAChR3, and mAChR5 subtypes in obese rats compared to that in lean ones. mAChRs mediate a wide range of functions peripherally and in the CNS. The five mAChR subtypes play a role in learning, memory, attention, and sensory-motor processing and are expressed differently in the brain [68,69,70]. However, we did not investigate their quantities.

The cholinergic alterations were accompanied by synaptic dysfunctions in the obese phenotype, with a reduction of SYP and synaptic vesicle protein SV2B in 20-week-old OZRs, both in the frontal cortex and in the hippocampus. These changes in the rat were associated with behavioral deficits. Cognitive decline was also reported by Bocarsly and coworkers in 2015 with decreased synaptic marker expression in the prefrontal and perirhinal cortex in DIO rats, accompanied by decreased dendritic spine density and changed microglia morphology [27]. Childhood metabolic disorders can impair cognitive development with abnormal synaptic function [71,72]. In addition, exposure to an HFD during the peak period of brain development can also alter neuroplasticity that links to eating disorders [73]. One study showed that longer periods of HFD feeding impair cognitive tasks associated with the hippocampus and reduce synaptic markers and increase microglia activation in the hippocampus [74]. On the contrary, another study reported no effect of long-term HFD on cognitive behaviors associated with the hippocampus [75]. It could be that obesity alone was not enough to compromise hippocampal structure and function. However, in combination with other complications such as chronic stress, it was sufficiently detrimental to impact hippocampal plasticity [76]. Interestingly, researchers found that restored cholinergic inputs and presynaptic synaptophysin contribute to the protective effects of physical running on spatial memory in aged mice [16].

## 5. Conclusions

The fact that the frontal cortex and hippocampus of OZRs are functionally compromised in cholinergic and synaptic activities provides new insight into how obesity can influence the cholinergic system and synaptic markers and, thus, the cognitive functions. Furthermore, the positive modulation of certain cholinergic and synaptic markers may be a possible therapeutic strategy for the treatment of obesity- and age-related cognitive dysfunction.

## Figures and Tables

**Table 1:** Primary antibodies used in Western blot (WB) and immunohistochemistry (IHC).

Antibodies	Company and Cat. No	Dilution WB	Dilution IHC
Vesicular acetylcholine transporter (VAChT)	Santa Cruz Biotechnology Cat. sc7717	1:500	1:100
Alpha7 nicotinic acetylcholine receptor (α7nAChR)	Santa Cruz Biotechnology Cat. sc5544	1:500	1:50
Muscarinic acetylcholine receptor subtype 1 (mAChR1)	Santa Cruz Biotechnology Cat. sc9106	1:500	1:50
Muscarinic acetylcholine receptor subtype 3 (mAChR3)	Santa Cruz Biotechnology Cat. sc7474	1:500	1:50
Muscarinic acetylcholine receptor subtype 5 (mAChR5)	Santa Cruz Biotechnology Cat. sc7479	1:500	1:50
Synaptophysin (SYP)	EMD Millipore Cat. MAB368	1:500	1:200
Synaptic vesicle glycoprotein 2A (SV2A)	Santa Cruz Biotechnology Cat. sc11939	1:200	1:50
Synaptic vesicle glycoprotein 2B (SV2B)	Santa Cruz Biotechnology Cat. sc11943	1:200	1:50
Synaptic vesicle glycoprotein 2C (SV2C)	Santa Cruz Biotechnology Cat. sc11946	1:200	1:50
Glyceraldehyde 3-phosphate dehydrogenase (GAPDH)	Sigma Aldrich Cat. G9295	1:5000	/

## Data Availability

Not applicable.

## Supplementary Materials

The following are available online at https://www.mdpi.com/article/10.3390/cells10102528/s1, Figure S1: Immunohistochemistry of synaptic vesicle glycoprotein 2A and synaptic vesicle glycoprotein 2C (SV2A and SV2C, respectively). Representative pictures of 20-week-old LZRs and OZRs in the frontal cortex (a) and hippocampus (b). V, VI: the fifth, sixth layers of the frontal cortex. P: pyramidal neurons in the CA1 and CA3 subfields of the hippocampus. SO: *stratum oriens*. SR: *stratum radiatum*. 40× magnification. Calibration bar: 25 µm.
