# Supplementary material for: Altered Brain Cholinergic and Synaptic Markers in Obese Zucker Rats
Source: Cells. 2021 Sep 24;10(10):2528. doi: 10.3390/cells10102528 (PMC8534069; doi:10.3390/cells10102528)
Supplement: Supplementary file 1 [file cells-10-02528-s001.zip › cells-1349514-supplementary.pdf]

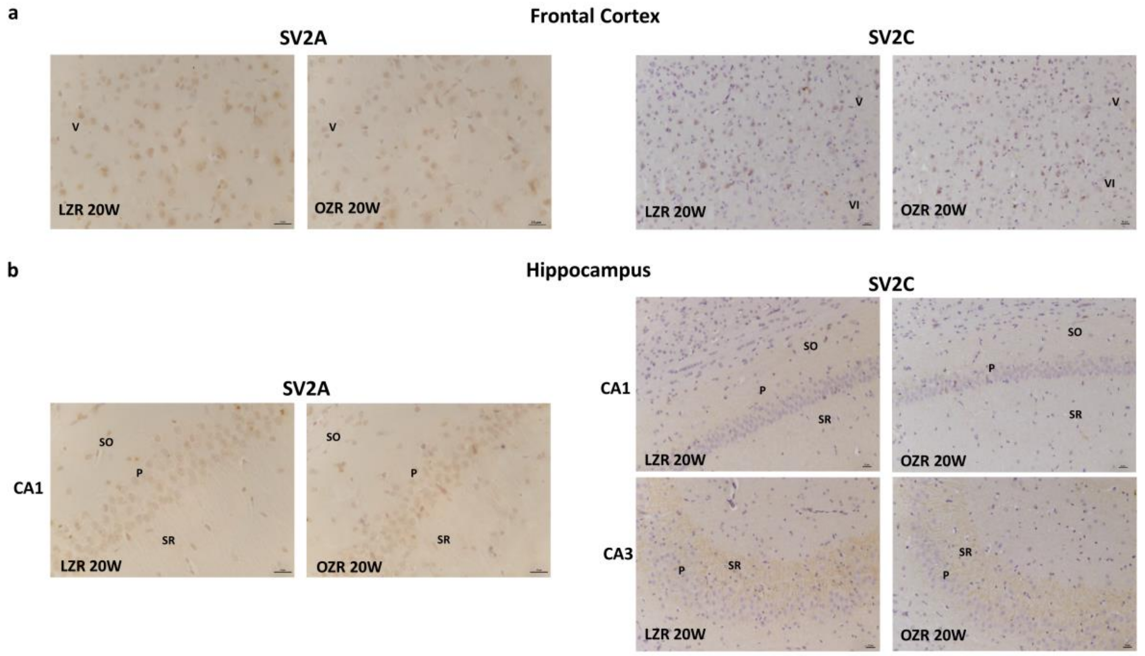


**Figure S1:** Immunohistochemistry of synaptic vesicle glycoprotein 2A and synaptic vesicle glycoprotein 2C (SV2A and SV2C, respectively). Representative pictures of 20 weeks old LZR and OZR in the frontal cortex (**a**) and hippocampus (**b**). V, VI: the fifth, sixth layers of the frontal cortex. P: pyramidal neurons in the CA1 and CA3 subfields of the hippocampus. SO: *stratum oriens*. SR: *stratum radiatum*. Magnification 40×. Calibration bar: 25 μm.
